# Patient’s Experience in Pediatric Primary Immunodeficiency Disorders: Computerized Classification of Questionnaires

**DOI:** 10.3389/fimmu.2017.00384

**Published:** 2017-04-05

**Authors:** Urs Mücke, Christian Klemann, Ulrich Baumann, Almut Meyer-Bahlburg, Xiaowei Kortum, Frank Klawonn, Werner M. Lechner, Lorenz Grigull

**Affiliations:** ^1^Department of Pediatric Hematology and Oncology, Hannover Medical School, Hannover, Germany; ^2^Department of Pediatric Surgery, Hannover Medical School, Hannover, Germany; ^3^Department of Pediatric Pulmonology, Hannover Medical School, Hannover, Germany; ^4^Department of Pediatrics, University Medicine Greifswald, Greifswald, Germany; ^5^Helmholtz Centre for Infection Research, Braunschweig, Germany; ^6^Ostfalia University of Applied Sciences, Wolfenbuettel, Germany; ^7^Improved Medical Diagnostics, IMD GmbH, Hannover, Germany

**Keywords:** primary immunodeficiency disease, data mining, diagnostic support, questionnaire, Colaizzi

## Abstract

**Introduction:**

Primary immunodeficiency disorders (PIDs) are a heterogeneous group of more than 200 rare diseases. Timely diagnosis is of uttermost importance. Therefore, we aimed to develop a diagnostic questionnaire with computerized pattern-recognition in order to support physicians to identify suspicious patient histories.

**Materials and methods:**

Standardized interviews were conducted with guardians of children with PID. The questionnaire based on parental observations was developed using Colaizzis’ framework for content analysis. Answers from 64 PID patients and 62 controls were analyzed by data mining methods in order to make a diagnostic prediction. Performance was evaluated by *k*-fold stratified cross-validation.

**Results:**

The diagnostic support tool achieved a diagnostic sensitivity of up to 98%. The analysis of 12 interviews revealed 26 main phenomena observed by parents in the pre-diagnostic period. The questions were systematically phrased and selected resulting in a 36-item questionnaire. This was answered by 126 patients with or without PID to evaluate prediction. Item analysis revealed significant questions.

**Discussion:**

Our approach proved suitable for recognizing patterns and thus differentiates between observations of PID patients and control groups. These findings provide the basis for developing a tool supporting physicians to consider a PID with a questionnaire. These data support the notion that patient’s experience is a cornerstone in the diagnostic process.

## Introduction

Primary immunodeficiency disorders (PIDs) in children are a group of more than 200 rare diseases presenting a wide spectrum of symptoms (1, 2). Although progress in genetic definition has been made, clinical diagnostics remains a challenging task for the general practitioner (GP) or pediatrician. Early diagnosis is of paramount importance because the delay leads to increased mortality, morbidity, and reduced quality of life (3, 4). Although the time to diagnosis varies, diagnostic delay is common (5–8).

Hence, several efforts have been made to support patient–provider communication and early referral to specialists. Diagnostic warning signs, education campaigns, and guidelines have been introduced in order to raise awareness and to shorten diagnostic delay (9, 10). However, Subbarayan et al. (11) state that existing tools do not work sufficiently and new approaches are requested (12). In this regard, patient’s medical histories offer clues for considering a PID. Previous studies have emphasized the importance of medical history taking as approximately 80% of the diagnoses could be established by careful history taking only (13, 14). However, these studies do not focus on PID. Due to the multitude of different immune defects and the highly variable clinical presentation, establishing the diagnosis of PID is particularly challenging. In some cases, the underlying rare condition mimics common diseases. In all cases, physician’s experience and background determine whether a referral and further testing are ordered (15). Before establishing a diagnosis, parents often recognize peculiarities. Yet, without an immunological expert at hand, it is difficult to put these observations into the context of a particular disease. Therefore, the question of how to decide which patient should receive further investigation gains supreme significance.

Prior to establishing a correct diagnosis, diagnostic errors occur in all medical fields (16, 17) and might be caused by various factors such as unusual or silent presentation of the disease, unavailability of expertise, or inadequate knowledge (18). Key findings suggest that patients should be enabled to tell their story appropriately, and doctors have to compensate an unavoidable lack of experience concerning rare diseases (19, 20).

Therefore, we introduce computerized analysis of parental observations collected in a novel questionnaire to provide additional support. Our aim was not to define a correct and specific diagnosis but to identify the need for timely referral to a PID specialist. Some studies already indicate the potential use of patient-centered questionnaires and data mining in diseases with a rather narrow spectrum of symptoms (21–23). To aid the discovery of unknown correlations or to derive recommendations for further action, computer-assisted analysis of huge amounts of data is useful (24). This targeted pattern analysis has already been established in internet search engines, banking, insurance, and marketing. It is used to make a prediction or to serve as an immediate alert function. In medicine such algorithms have been applied successfully in different contexts (25, 26).

We hypothesized that pre-diagnostic experiences of PID patients could be used to develop a questionnaire. Such a questionnaire should distinguish different patient cohorts using data mining classifiers. This investigation could establish a basis to develop a computerized diagnostic support tool.

## Materials and Methods

At first, patient-centered semi-structured interviews with guardians of children with a confirmed PID were conducted. The study protocol was approved by the ethics committee of Hannover Medical School and written informed consent was obtained from each guardian. An overview of the diseases included in the interview process is provided in Table 1.

**Table 1:** **Spectrum of diseases in the interviews: 12 interviews with parents of children suffering from different diseases were conducted**.

Interview	Category	Disease
A	Predominantly antibody deficiencies	CVID
B	Predominantly antibody deficiencies	CVID
C	Well-defined syndromes with immunodeficiency	Ataxia teleangiectasia
D	Predominantly antibody deficiencies	CVID
E	Complement deficiencies	C2 deficiency
F	Combined immunodeficiencies	Cernunnos
G	Undefined immunodeficiency	Combined immunodeficiency with different cytopenia
H	Well-defined syndromes with immunodeficiency	Nijmegen breakage syndrome
I	Congenital defects of phagocyte number, function, or both	X-linked chronic granulomatous disease
J	Undefined immunodeficiency	Undefined severe immunodeficiency
K	Predominantly antibody deficiencies	CVID
L	Well-defined syndromes with immunodeficiency	Nijmegen breakage syndrome

*Most common diagnosis was common variable immunodeficiency (CVID, *n* = 4)*.

**Figure 1:**
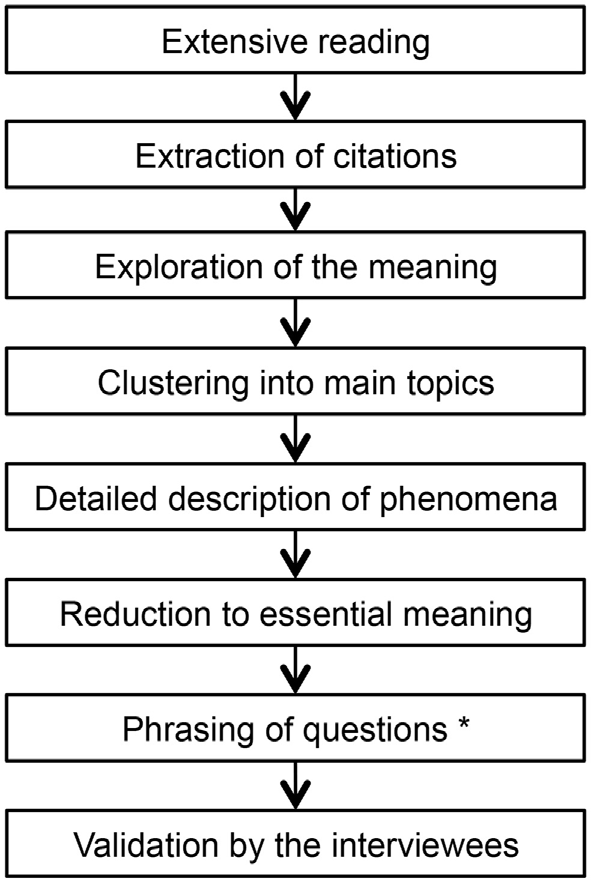
**Colaizzi’s framework contains defined steps for standardized content analysis**. Modification: “phrasing of questions” was inserted for study purpose.

The selection of interview partners followed predefined principles: sufficient speech comprehension, written consent, confirmed PID, children’s age 0–18 years, majority of the eight IUIS-categories (27) should be represented by at least one interview, inclusion of further interviewees until theoretical saturation (28). Interviewers used a guideline for standardized procedure and a uniform beginning (what did you observe regarding your child’s health and general development before the doctors could finally tell you that your child suffers from a PID?). Each interview was analyzed using Colaizzi’s framework with respect to phenomena experienced by parents in the pre-diagnostic period (see Figure 1). This standardized procedure contains seven defined steps and is well established in social sciences for content analysis (29). The results of the analysis were documented in a table containing a separate column for each step. The phenomena were sorted depending on occurrence in the interviews in order to integrate relevant observations in the questionnaire. For study purpose, we added a step for question generation (see Figure 1).

Members of the study group used the results to draft questions. The parental point of view and the choice of words were integrated in the development of the questionnaire. All questions were summarized in a question pool for further selection. Each question received an identification number for retraceability to its origin. The total number of questions was systematically reduced following predefined requirements: each phenomenon relevant for the pre-diagnostic period was represented by at least one item, and the most relevant phenomena were incorporated by additional questions. Duplicates were canceled, a pretest for comprehensibility, consensus in the study group, and expert opinion were integrated. The full resulting questionnaire consequently reflects the pre-diagnostic experience of parents with a child affected by PID (Table S1 in Supplementary Material).

### Distribution of the Questionnaire

To generate a data set, the questionnaire was distributed to patients who visited the Immunological Outpatient Clinic of the Hannover Medical School in 2013 and 2014 for regular appointment. All patients had an established diagnosis of PID. An additional online version was accessible for members of the patient group of PID in Germany [Deutsche Selbsthilfe Angeborene Immundefekte (DSAI)] with a special access code. As a control group, we randomly collected questionnaires from guardians of healthy children and children who were hospitalized due to a disease other than PID.

### Data Mining Methods

Statistical software libraries offer different computer-based methods to analyze and classify data. Most of these methods are variations of main statistical concepts like vector space methods or artificial neural networks. The tool under discussion uses support vector machines, random forests, logistic regression (LR), naive Bayes classifiers, linear discriminant analysis and nearest neighbor classifiers (30). Classifiers were trained and tested with questionnaires processed in numeric table format. A fusion algorithm combined the different predictions made by each single classifier to one final decision (31). Validation of the system was performed in two steps. First, the diagnostic accuracy was challenged using two sets of questionnaires: children with PID and healthy children. In a second step, the system had to distinguish between questionnaires of PID children and a combination of randomly chosen healthy and sick children with different diseases, e.g., severe bronchitis, brain tumor, cystic fibrosis, and ulcerative colitis (full spectrum, see Table S2 in Supplementary Material). On both levels, validation was performed by *k*-fold stratified cross-validation. In addition, we analyzed questions concerning their contribution to the correct classification based on the *p*-Value for the coefficients in LR, i.e., with the null hypothesis that the coefficient for the question is 0, meaning that the corresponding question does not contribute significantly to the prediction based on LR.

## Results

### Core Phenomena Were Revealed through Frequent Parental Observations in Standardized, Semi-Structured Interviews

The analysis of the interviews using Colaizzi’s framework revealed major themes such as chronological characteristics of infections, parental perception of infections, susceptibility to infections in everyday life, infections of the respiratory tract, and effectiveness of antibiotic treatment (Table S3 in Supplementary Material). These were expressed in words and concepts by the target group.

### Generation of a 36-Item Questionnaire by Standardized Content Analysis

**Figure 2:**
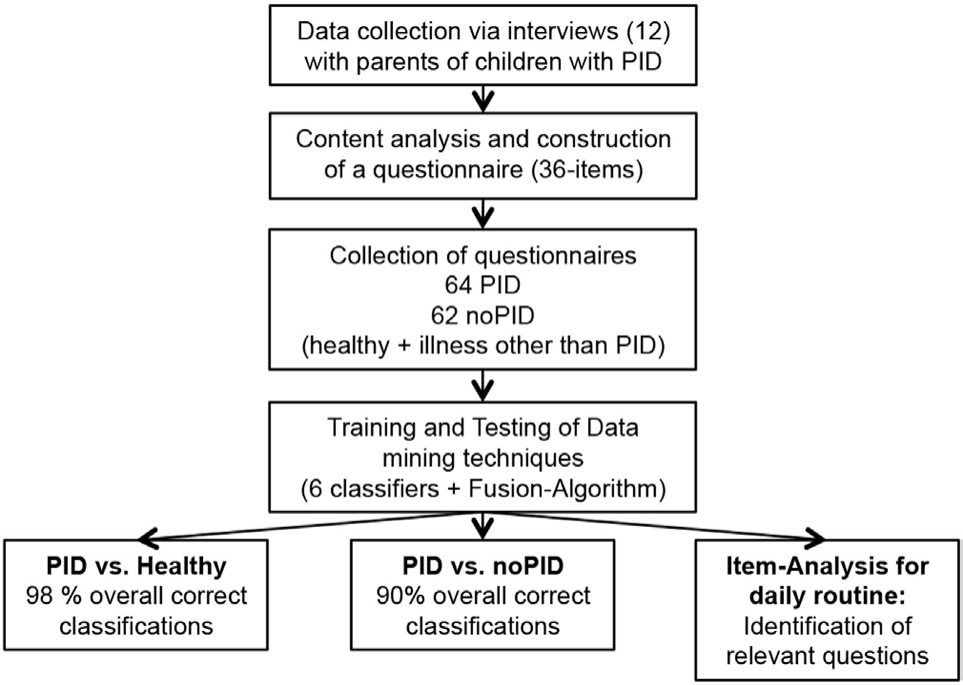
**Flow chart of the study procedure**. After conduction of 12 interviews with parents of primary immunodeficiency disorder (PID) patients, content analysis was used to develop a 36-item questionnaire. The novel questionnaire was utilized to collect data for training and testing of data mining techniques concerning classification approaches resulting in sensitivity up to 98%.

Based on major themes and quotations, 186 preliminary questions were phrased to represent the parental perspective (not shown). For the final version, the questions were systematically reduced (Table S1 in Supplementary Material). Reasons for exclusion were: duplications in form and content, irrelevance for the pre-diagnostic period, and incomprehensibility. Modifications were discussed with experienced immunologists and approved by consensus of the study group. The final questionnaire was subsequently completed by 126 parents. A flow chart of the process is shown in Figure 2.

**Figure 3:**
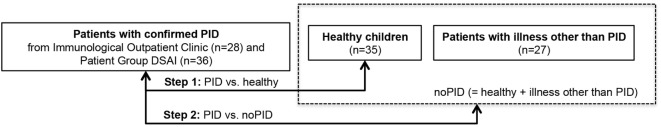
**Data source of questionnaires ordered by category showing stepwise analysis approach**. Questionnaires were distributed to three groups and subsequently used for data mining training and test. Test of classification skills was performed in step 1 [primary immunodeficiency disorders (PIDs) vs. healthy] and step 2 (PID vs. no PID). No-PID contains questionnaires of healthy children and children with illness other than PID.

Here, the final questionnaire was completed by parents from different patient collectives. The resulting raw data set consisted of 64 questionnaires from children with PID and 62 questionnaires without PID (35 healthy + 27 patients with illness other than PID, see Figure 3). The most common diagnoses in the PID group were common variable immunodeficiency disorders (CVID) (*n* = 11) and agammaglobulinemia (*n* = 10) (see Table 2).

**Table 2:** **The four most frequent diagnoses of children with primary immunodeficiency disorders (PIDs): the four most frequent diagnoses of a total of 64 questionnaires received from PID patients**.

Diagnosis	*n*
Common variable immune deficiency (CVID)	11
Agammaglobulinemia	10
Syndromes with recurrent fever (TRAPS + PFAPA)	6
Chronic granulomatous disease	5

*TRAPS, TNF receptor-associated periodic syndrome; PFAPA, periodic fever with aphthous stomatitis, pharyngitis, and adenitis*.

Diagnoses in the illness-other-than-PID group were e.g., acute lymphatic leukemia, colitis ulcerosa, cystic fibrosis, and chronic renal failure (Table S2 in Supplementary Material).

### Identification of Suspicious Answer Patterns by Data Mining Shows a Sensitivity of up to 98%

99 individuals answered the questionnaire for step 1. This group consisted of 64 individuals with PID and 35 healthy children serving as a control group (see Figure 2). A 11-fold stratified cross validation showed an overall sensitivity of 98%. Regarding the group of children with PID, 63 of 64 (98%) received the correct diagnosis, and 34/35 of the controls were classified correctly. In total, 97/99 individuals received the correct diagnosis in the cross-validation. Subsequently, 27 questionnaires of randomly chosen children with different diseases were added to the data set (see Figure 3). A 21-fold stratified cross-validation was performed to examine diagnostic sensitivity.

In this group (PID patients compared to healthy + illness other than PID), 113/126 (90%) of the questionnaires were classified correctly. In detail, 61/64 individuals with PID received the correct diagnosis, but only 52/62 individuals were correctly classified as “no-PID.”

### Analysis Discovered Questions Significant for Differentiation

The analysis of the 36 questions, using *p*-Value computation, revealed 32 questions which contributed to the correct classification significantly (*p* < 0.05). Questions with highest significance in step 2 (PID vs. healthy + illness other than PID) were

Q1 (Did your child suffer from ill health constantly?)
Q31 (Is it true that your child’s infections lasted longer than the ones of other children?)
Q32 (Is it true that your child was treated with antibiotics regularly?)
Q35 (Is it true that the doctors could not tell what your child was suffering from?)

### Three-dimensional Visualization of Answer Patterns Differentiates Healthy from Sick Children

**Figure 4:**
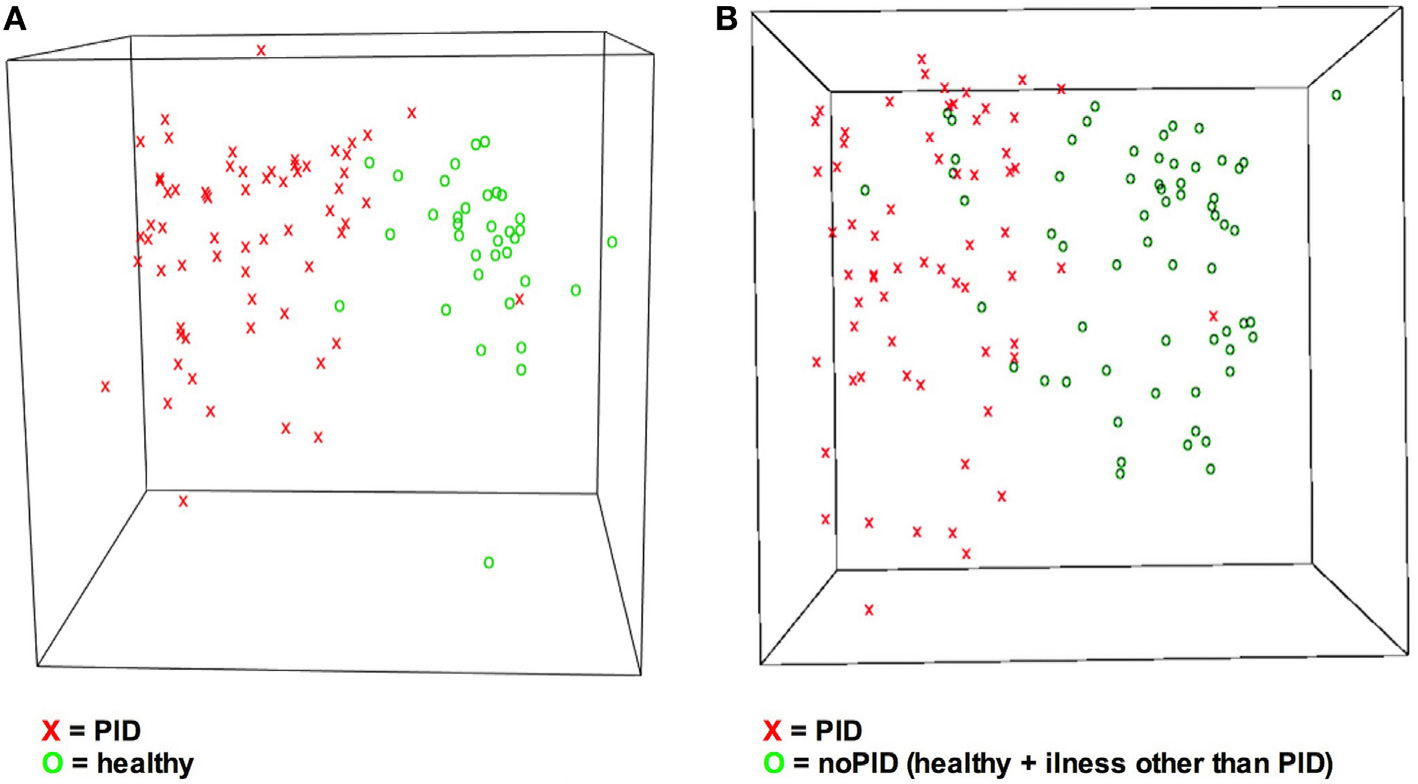
**Visualization of answers indicates different answer pattern**. Answer pattern can be visualized by the dimension reduction technique Sammon mapping. X = primary immunodeficiency disorder (PID), O = control. **(A)** Classes PIDs vs. healthy are easy to distinguish. **(B)** Due to increased heterogeneity it is more difficult to discriminate classes PID vs. no-PID. No-PID contains questionnaires from healthy children and children suffering from illnesses other than PID.

In terms of a classification problem the answer pattern classification with 36 items consists of 36 dimensions as 36 questions were answered. Based on Sammon mapping, a three-dimensional representation of the data was generated (see Figure 4) (32). It visualizes the questionnaires concerning the correct classification, and illustrates the general principle of distinguishing two groups of patients. Comparing step 1 and step 2 of the study (Figures 4A,B) more overlapping of the cohorts is shown in Figure 4B. Overlapping questionnaires can represent misdiagnosed cases.

## Discussion

The diagnosis of PID presents a challenge to pediatricians due to rare incidence and unspecific symptoms, resulting in delayed referral and negative outcome for patients and parents (3, 4). The pre-diagnostic experience of patients and their families is not sufficiently integrated into the diagnostic process. The study at hand indicates that a novel combination of a questionnaire and data mining techniques provides the means to identify suspicious answer patterns.

Our data show that patient interviews are a feasible tool to generate questionnaires. In order to improve the quality of the questions, parental interviews were obtained to collect observations from the pre-diagnostic time. Colaizzi’s method is widely applied in nursing science and qualitative research and has proven useful to derive knowledge from interviews (33, 34). It appears to be useful to collect personal experience and to mine it for the generation of a questionnaire (35). The data generated in this study supports the impact of qualitative research in the diagnostic process (36). Our data give important insights into the parents’ perceptions prior to diagnosis as opposed to clinical focus, and likewise reasons for the diagnostic delay could be exposed. An advantage of Colaizzi’s framework is the standardized procedure, which makes the connection between citation and generated question retraceable (37).

In our approach, we combined six classifiers and added a fusion algorithm to increase sensitivity. Our combination showed a sensitivity of 84–98%. In comparison of step 1 and step 2, sensitivity decreased when adding sick children to healthy controls. The overall sensitivity of 90% in step 2 still underscores the promising performance of the techniques piloted here. The application of a fusion algorithm improved the diagnostic quality of the tool. Recently, the concept of questionnaires and data mining was successfully piloted by Rother et al. (23). They applied related techniques on pulmonary diseases and achieved encouraging results.

The combination of a questionnaire and data mining has already been tested in diagnosing gastroesophageal reflux disease (GERD) (21). The tool differentiates between complaints caused by GERD and other dyspeptic disorders. In contrast to our study, Horowitz et al. used a shorter questionnaire (15 vs. 36 items), did not use extensive interviews to accumulate observations, and applied less data mining techniques.

It is important to emphasize that the process of professional medical history taking is neither intended nor suitable to be replaced by questionnaires. In fact, history taking might be improved because the diagnostic tool highlights those questions, which data mining suggested to be of high diagnostic relevance. Physicians can clarify the past medical history and receive additional hints (38–40). The acceptance of diagnostic questionnaires has already been proven (41).

Some experts stress the notion that more awareness for PID is needed among physicians in tertiary hospitals (11). Yet, Lankisch et al. (42) suggest focusing on primary care physicians and pediatricians without profound experience in PID. Lankisch et al. provided a modified catalog of warning signs and achieved improved results for detecting children with PID compared to classic warning signs (12, 42). However, they underlined the need for improvements in the diagnostic pathway in order to detect preferably 9 out of 10 children suffering from PID. The tool piloted here might help to fill this gap despite the preliminary character of the study as well as its limitations. Different paper-based questionnaires are nowadays used on occasion in outpatient clinics to collect data about patients’ medical history. They are based on expert opinion, most are not empirically verified, and they are interpreted manually deriving hints depending on examiners expertise. In contrast, our tool focuses on the patients or guardians perspective, can be statistically reviewed, is extendable, improved by adding more data, and it works independently from the doctor’s experience.

Parts of our questionnaire reflect current guidelines (43). Additionally, the questions with high impact for the diagnosis PID share similarities to the classical “warning signs” but also differences. The usage of antibiotics is included in both tools. Simple questions like Q1 (did your child suffer from ill health constantly?) and Q36 (is it true that your child was absent from school/pre-school/kindergarten due to sickness more often than other children?) are solely derived from parental experience and revealed an important contribution to correct classification. In contrast to an analysis by Subbarayan et al. (11), a positive family history was not significant for finding the correct diagnosis in our cohort. This result supports a presumption by Brodszki et al. (44) who states that the degree of consanguinity in the collective is essential. Kallus (45) underlines that a specific attribute has to be represented in a sample often enough to reach sufficient discriminatory power. For this reason, a questionnaire containing more than 10 items increases the probability to catch sufficient observations. Nevertheless, it is not a single question that makes the difference. It is the combination of several questions with different contributions to the correct classification that makes diagnostic hints possible.

Our study has several limitations: first of all, as it is a study for proof of concept, only a narrow spectrum of diagnoses was included in the interview phase. Likewise, only a small part of all known PID was represented. Second, the number of patients and controls is limited. Nevertheless, cross-validation supports the results which need re-evaluation in a prospective trial. Besides, diagnostic support was also possible for those diagnoses not included in the interviews indicating the plasticity of the system. Third, using a written questionnaire limits the usage of a tool. For example, it requires enough language comprehension. The questionnaire was generated and evaluated in German. Today, the transfer into different languages and cultures and reproduction of the excellent results is unproven. In the era of multicultural societies, a universal comprehensibility independent of language ability would be desirable (46). Tablet computers, which have already been positively evaluated in other areas could be used to reduce the effort for completion of the questionnaire and its interpretation (47, 48). Furthermore, possible online adaptions of paper versions already proofed usability in other areas (49). A further limitation is related to the selection of controls. The cohorts do not reflect the regular spectrum of patients seen by GP or pediatricians. Ideally, this study should have incorporated a control group representative of the day-to-day population of GP and/or general pediatricians. Controls presenting with symptoms suspicious for a PID would have answered a questionnaire prior to the referral to an immunologist. Due to limited resources, this evaluation will be part of a future trial based on this pilot proof of concept. Thus, further prospective studies with patients presenting PID-like symptoms are needed to verify our approach.

Taken together, we successfully piloted a questionnaire-based tool intended to classify different patient cohorts.

A diagnostic tool for detection of children with PID could be used in different settings: the GP or the pediatrician could hand it to parents with children showing recurrent infections to reassure the need for further diagnostics. The questionnaire could be answered on a tablet computer while sitting in the waiting area. A diagnostic suggestion would only be presented to the physician who could include the computerized diagnostic suggestion into the diagnostic workflow and use eye-catching answers for clarifying.

A series of five clinical cases as an example for the prospective classification process is given in Table S4 in Supplementary Material. Extensive investigation of user friendliness is part of a prospective evaluation. The principles applied in this study might also be extended to other groups of rare diseases beyond PID.

## Author Contributions

UM and LG conceptualized the study, conducted the interview, analyzed transcripts, and wrote the manuscript. WL, FK, and XK performed all data mining. CK, UB, and AM-B supervised the clinical process. All authors discussed the results and commented on the manuscript.

## Conflict of Interest Statement

WL, FK, and LG are co-founders of IMD (IMD GmbH, Deutschland). The other authors have no conflicts of interest to disclose. The handling editor declared a past co-authorship with one of the authors UB and states that the process nevertheless met the standards of a fair and objective review.

## Abbreviations

PID, primary immunodeficiency disorder; SVMs, support vector machines, RFs, random forests; LR, logistic regression; NB, naive Bayes classifiers; LD, linear discriminant; NNs, analysis and nearest neighbor classifiers.
