# Supplementary material for: Patient’s Experience in Pediatric Primary Immunodeficiency Disorders: Computerized Classification of Questionnaires
Source: Front Immunol. 2017 Apr 5;8:384. doi: 10.3389/fimmu.2017.00384 (PMC5380667; doi:10.3389/fimmu.2017.00384)
Supplement: Supplementary file 1 [file Data_Sheet_1.PDF]

Suppl. Table 1. 36-Item Questionnaire (Original in German)

**No.**	**Question**
1	Did your child suffer from ill health constantly?
2	Did your child eat and drink sufficiently?
3	Do you think that further examinations (e.g. blood test) should have been done in order to verify your observations concerning your child’s health?
4	Did your child’s illness already appear “normal” to you?
5	Did your child regain full health between periods of illness?
6	Did your child’s illness repeatedly affect the same body regions (e.g. always the ear, always the lungs, …)?
7	Did your child look slim/frail?
8	Did your child suffer from diarrhea?
9	Did you regularly notice unusual breathing sounds?
10	Did your child suffer from constant coughing?
11	Did your child have swollen lymph nodes?
12	Did your child suffer from high fever without the cause for the fever being found?
13	Did you notice a regular white-ish film in your child’s mouth that was difficult to wipe off?
14	Did your child have a small head compared to other children?
15	Would you say your child was fit enough for sports and physical exercise?
16	Did antibiotics work satisfactorily when your child was treated with them?
17	Do any known immunodeficiencies run in your family?
18	Are there any known cases of children’s deaths in your family for which no clear cause could be found?
19	Would you say that exceptionally many members of your family have been diagnosed with cancer in the past?
20	Did you have to take your child to hospital?
21	Did doctors find any unusual blood values in your child (e.g. changes in blood count)?
22	Did your child suffer from pneumonia?
23	Did you have to consult a doctor due to recurring earaches?
24	Did your child suffer from meningitis?
25	Did you have to use antipyretic medication (per month)?
26	Did your child suffer from fevers that could not or not really be lowered by treatment with medication?
27	Is it true that you repeatedly switched doctors because the reasons for your child’s health issues could not be found?
28	Is it true that your child’s health issues increased consistently?
29	Is it true that everyday incidents (like not being dressed warmly enough) sufficed for your child to fall ill?
30	Is it true that your child’s illnesses repeatedly showed the same or similar patterns?

31 Is it true that your child’s infections lasted longer than the ones of other children?

32 Is it true that your child was treated with antibiotics regularly?

33 Is it true that you consulted a doctor repeatedly due to similar or identical health issues?

34 Is it true that your child has always been a bit smaller than other children?

35 Is it true that the doctors could not tell what your child was suffering from?

36 Is it true that your child was absent from school/pre-school/kindergarten due to sickness more often than other children?

Suppl. Table 2. Diseases used for comparison with PID in step 2 (PID vs. noPID)

**Number**	**Disease**
1	Undefined weakness
2	Acute lymphatic leukemia
3	Acute lymphatic leukemia
4	Periodic fever without diagnosis of PID
5	Ulcerative colitis
6	Severe bronchitis
7	Multiple allergies with urticaria without diagnosis of PID
8	Solitary mild Infection with fever
9	Cystic fibrosis and diabetes
10	Progressive glomeruolonephritis
11	Unspecific symptoms
12	Multiple Allergies without diagnosis of PID
13	Cystic fibrosis
14	Leukemia
15	Brain tumor
16	Cystic fibrosis
17	Chronic renal failure
18	Hemolytic uremic syndrome
19	Burkitt lymphoma
20	Denys-Drash-Syndrom
21	Polycystic kidney disease
22	Acute lymphatic leukemia
23	Lymphoma
24	Chronic bronchitis without diagnosis of PID
25	T-Cell Acute lymphatic leucemia
26	Epilepsy
27	Renal dysplasia

Suppl. Table 3. Core phenomena derived from parental observations (Original in German)

Category Title	Interview reference
**Consultation**	
Consultations from the parental point of view	A,B,F,G,I,K,L
Tentative diagnoses	C,E,F,K,L
Alarm signals for acute illness	E,H,L
**Therapy**	
Effectiveness of antibiotic treatment	A,H,I,J,L
Antibiotic treatment	A,G,J,K
Use of immunoglobulin	F,L
**Behavior**	
… of the parents	
Evaluation of the own behavior by the parents	A,I
Food intake	F,K,L
Milestones of the development from the parental perspective	C,F,J
**Infections**	
Chronologic characteristics	B,C,E,F,G,H,K,L
Pathogens	E
Susceptibility to infections	C,D,F,G,L
Otorhinolaryngology	F,H,I,K
Airway	F,G,I,J,L
Sepsis or Meningitis	H,L
**Attendant symptoms**	
Fever	D,E,F,G,H,J,L
Gastrointestinal	F,I
**Perception of the disease**	
… by professionals	A,E,F,H,K,L
… by parents	
Adaption to the disease	A,C,D
Training of parents in self diagnostic	A,H,L
… of infections by the parents	A,B,D,F,G,H,K,L
Explanatory model	C,G,H,K
Health from the parental perspective	C,E,L
Subjective feeling in daily life	D,K

**Appearance of the child**	
Weight	A,F,K,L
Lymphnodes	B
Musculoskeletal system	C
Growth	F,H,I,L
Skin	E,F,G,J,K
Cognitive development	L
**Diseases in the family**	
Immunodeficiencies	G
Unclear cases of death	E,F,J
Cancer	L

Suppl. Table 4. Example of prospective clinical cases

Patient No	Symptoms	Decision by clinician	Decision by data mining	diagnosis
1	3 years old female with wound healing disorder, multiple abscess,peritonitis with Staph. Aureus	PID as far as possible excluded	no PID	CGD excluded, PAD improbable due to normal immunoglobulins
2	5 years old male with fever (max 40 °C) every 3-4 days, antifebrils with little effect	PID probable	PID	PFAPA
3	14 years old male, pediatrician recommends further tests because of orofacial herpes twice monthly	PID improbale	no PID	Immunoglobulins normal, antibodies from vaccination, NK- and T-cellfunction regular, subpopulations normal
4	9 years old male, Mycobacterium avium in cervical lymph nodes, fistula formation, nail mycosis, chronic squamous erythema at extremities	PID very likely	PID	functional deficient STAT4 signal transduction pathway
5	10 years old female, infection of the anterior tibialis lodge with salmonella typhimurium, recurrent abscessus	rather not a PID	no significant differentiation	lower total B-Cell count, lower IgM Memory cells, normal class switched B-Cell count. T-cells and NK-cells regular, immunoglobulins normal, complement diagnostics unobtrusive
